# [^64^Cu]‐labelled trastuzumab: optimisation of labelling by DOTA and NODAGA conjugation and initial evaluation in mice

**DOI:** 10.1002/jlcr.3287

**Published:** 2015-04-24

**Authors:** Christina Schjoeth‐Eskesen, Carsten Haagen Nielsen, Søren Heissel, Peter Højrup, Paul Robert Hansen, Nic Gillings, Andreas Kjaer

**Affiliations:** ^1^ Department of Clinical Physiology, Nuclear Medicine and PETRigshospitaletCopenhagenDenmark; ^2^ Cluster for Molecular ImagingUniversity of CopenhagenCopenhagenDenmark; ^3^ Department of Biochemistry and Molecular BiologyUniversity of Southern DenmarkOdenseDenmark; ^4^ Department of Drug Design and PharmacologyUniversity of CopenhagenCopenhagenDenmark

**Keywords:** DOTA, NODAGA, trastuzumab, PET

## Abstract

The human epidermal growth factor receptor‐2 (HER2) is overexpressed in 20–30% of all breast cancer cases, leading to increased cell proliferation, growth and migration. The monoclonal antibody, trastuzumab, binds to HER2 and is used for treatment of HER2‐positive breast cancer. Trastuzumab has previously been labelled with copper‐64 by conjugation of a 1,4,7,10‐tetraazacyclododecane‐1,4,7,10‐tetraacetic acid (DOTA) chelator. The aim of this study was to optimise the ^64^Cu‐labelling of DOTA‐trastuzumab and as the first to produce and compare with its 1,4,7‐triazacyclononane, 1‐glutaric acid‐5,7 acetic acid (NODAGA) analogue in a preliminary HER2 tumour mouse model. The chelators were conjugated to trastuzumab using the activated esters DOTA mono‐*N*‐hydroxysuccinimide (NHS) and NODAGA‐NHS. ^64^Cu‐labelling of DOTA‐trastuzumab was studied by varying the amount of DOTA‐trastuzumab used, reaction temperature and time. Full ^64^Cu incorporation could be achieved using a minimum of 10‐µg DOTA‐trastuzumab, but the fastest labelling was obtained after 15 min at room temperature using 25 µg of DOTA‐trastuzumab. In comparison, 80% incorporation was achieved for ^64^Cu‐labelling of NODAGA‐trastuzumab. Both [^64^Cu]DOTA‐trastuzumab and [^64^Cu]NODAGA‐trastuzumab were produced after purification with radiochemical purities of >97%. The tracers were injected into mice with HER2 expressing tumours. The mice were imaged by positron emission tomography and showed high tumour uptake of 3–9% ID/g for both tracers.

## Introduction

The application of monoclonal antibodies (mAbs) in oncology continues to increase and includes diagnostic imaging and radionuclide therapy.^1^ MAbs are often labelled by conjugating a bifunctional chelator to which a radiometal can bind. The affinity of the mAb should be maintained during these modifications.^2^ A variety of chelators are available for conjugation, but their application depends on the radiometal used.^3^ The radiometal chelator‐complex should have high *in vivo* stability to avoid distribution of free metal to normal tissue.^4^ The large size of mAbs prevents direct renal elimination, and mAbs are instead metabolised to smaller molecules, which then can be excreted. The slow metabolism leads to long serum half‐life of mAbs and can cause high background concentrations in imaging and high radiation doses to non‐target organs during radionuclide therapy.^5^, ^6^, ^7^ Better target to background ratios in imaging can be obtained by labelling mAbs with long‐lived radioisotopes and image 24–72 h after administration.^8^ A useful isotope for mAb imaging is the positron‐emitting radionuclide copper‐64, with a half‐life of 12.7 h and positron range suitable for positron emission tomography (PET) imaging.^9^ Copper‐64 can coordinate to a variety of chelators but some require harsh conditions incompatible with mAbs.^8^, ^10^

Trastuzumab, also known as Herceptin, is a humanised IgG1 mAb that targets the human epidermal growth factor receptor‐2 (HER2).^11^ Trastuzumab was the first mAb to be approved by the US Food and Drug Administration for targeting HER2 and has been used for treatment of HER2‐positive breast cancer since 1998.^12^ HER2 is overexpressed in 20–30% of breast cancer and leads to increased cell proliferation, cell growth and cell migration.^13^, ^14^ The binding of trastuzumab to HER2 inhibits tumour growth, making it useful for treatment in combination with chemotherapy and radiation therapy.^15^ HER2 overexpression is routinely determined by immunohistochemistry or fluorescence *in situ* hybridization of a tumour biopsy. These methods are not always reliable, and discordance in HER2 expression between primary and metastatic lesions has been found in some cases. A molecular imaging method to determine HER2 expression more reliably is thus needed.^16^

Trastuzumab was initially labelled with indium‐111, and primary and metastatic lesions could be visualised in mice by imaging with single‐photon emission computed tomography (SPECT).^17^ [^111^In]‐Labelled trastuzumab was studied in humans, and HER2‐positive tumour lesions and new tumour lesions were detected. SPECT has limitations regarding spatial resolution and sensitivity in deep tissue.^18^ To optimise HER2 detection further, PET has been used with zirconium‐89 labelled trastuzumab.^19^, ^20^, ^21^ [^89^Zr]‐Labelled trastuzumab accumulated in HER2‐positive tumours in mice and was further evaluated in humans. Tumour uptake was high in patients with metastatic breast cancer, and HER2‐positive tumour lesions could also be detected in liver, lung, bone and brain.^21^ Labelling biomolecules with zirconium‐89 is useful because of its long half‐life of 3.3 days.^22^ The radiation dose to patients from [^89^Zr]‐labelled trastuzumab is 2.5 times higher than imaging with 2‐deoxy‐2‐[^18^F]fluoro‐D‐glucose. Thus, a radionuclide with a shorter half‐life, such as copper‐64, might be a better choice for HER2 imaging.^21^

Niu *et al*. used [^64^Cu]DOTA‐trastuzumab to monitor HER2 degradation with therapy treatment in a human ovarian cancer model in mice.^23^ The tumour uptake was significantly different between the control and the treatment groups, 33.9 ± 7.82 versus 11.74 ± 4.23% ID/g 24 h post injection. [^64^Cu]DOTA‐trastuzumab also accumulated in HER2 positive tumours in a study with non‐small cell lung cancer in mice.^24^ Ferreira *et al*. compared the chelators 1‐oxa‐4,7,10‐triazacyclododecane‐4,7,10‐triacetic acid (oxo‐DO3A) and 3,6,9,15‐tetraazabicyclo[9.3.1]pentadeca‐1(15),11,13‐triene‐3,6,7‐triacetic acid (PCTA) to DOTA in the ^64^Cu‐labelling of trastuzumab.^8^ The labelling of oxo‐DO3A‐trastuzumab and PCTA‐trastuzumab was quicker and afforded higher radiochemical yields compared with DOTA‐trastuzumab. Higher stability in mouse serum was also seen compared with DOTA. *In vivo* studies in mice showed higher tumour uptake of [^64^Cu]oxo‐DO3A–trastuzumab and [^64^Cu]PCTA‐trastuzumab than [^64^Cu]DOTA‐trastuzumab 24 ‐h post injection. At 40‐h post injection, all three compounds had similar uptakes and no significant difference in biodistribution was observed. In a PET human HER2 study, [^64^Cu]DOTA‐trastuzumab accumulated in HER2‐positive tumours and metastatic brain lesions could also be visualised.^25^

In this study, the copper‐64 labelling of DOTA conjugated trastuzumab was optimised. Due to the possibility of copper‐64, dissociation from the DOTA chelator *in vivo* an alternative chelator, NODAGA, was also investigated for comparison. This is the first report on preparation and evaluation of [^64^Cu]NODAGA‐trastuzumab.

## Experimental

### General information

Trastuzumab (Herceptin®) was purchased from Roche and used without purification. 2,2′,2″‐(10‐(2‐((2,5‐dioxopyrrolidin‐1‐yl)oxy)‐2‐oxoethyl)‐1,4,7,10‐tetraazacyclododecane‐1,4,7‐triyl)triacetic acid (DOTA‐NHS) and 2,2′‐(7‐(1‐carboxy‐4‐((2,5‐dioxopyrrolidin‐1‐yl)oxy)‐4‐oxobutyl)‐1,4,7‐triazonane‐1,4‐diyl)diacetic acid (NODAGA‐NHS) were purchased from CheMatech (Dijon, France). PD MiniTrap Columns and PD‐10 Desalting Columns were purchased from GE Healthcare. All aqueous solutions used for conjugation and radiolabelling were made with TraceSELECT® Water from Sigma‐Aldrich. Ethylenediaminetetraacetic acid (EDTA), salts for buffers, acetonitrile, trifluoroacetic acid (TFA), sinapinic acid and Eppendorf LoBind tubes were also purchased from Sigma‐Aldrich. Formic acid was purchased from Merck Millipore and Technisolv® ethanol from VWR. [^64^Cu]CuCl_2_ was produced on a PETtrace cyclotron (GE Healthcare) by the ^64^Ni(p,n)^64^Cu nuclear reaction at Hevesy Laboratory, Risoe, Denmark. HPLC analysis was performed on a Gilson HPLC with a Dionex UV lamp (UVD170U) and a Scansys radiodetector. A Phenomenex Yarra SEC‐2000 column (3 μ, 300 × 7.8 mm) was used with 0.1‐M phosphate buffer pH 7 as mobile phase at a flow rate of 1 ml/min. Thin‐layer chromatography (TLC) was performed using a Scan‐Ram radio‐TLC scanner detector (LabLogic) or a Packard Instant Imager with instant TLC strips (Agilent Technologies) eluted with a 0.1‐M EDTA solution in phosphate buffer (pH 7). Antibody concentrations were determined using a NanoDrop 1000 from Thermo Scientific. Matrix‐assisted laser desorption ionization time‐of‐flight mass spectrometry (MALDI‐TOF MS) was performed using an Ultraflextreme™ MALDI‐TOF/TOF mass spectrometer (Bruker Daltonik GmbH). All spectra were recorded using linear mode and positive polarity. The singly charged ion, doubly charged ion, and singly charged dimer of bovine serum albumin (M_r_ 66,430 Da) were used for a three‐point calibration.

### DOTA/NODAGA conjugation

To a solution of trastuzumab in 0.1‐M borate‐buffered saline (10 mg/ml) was added 5, 20 or 100 equivalents of DOTA‐NHS ester or NODAGA‐NHS ester in 0.1‐M borate‐buffered saline. The mixture was left at 4°C for 20 h. Excess chelator was removed by size exclusion chromatography with a PD‐10 column using 0.1 M NaOAc (pH 5.64) as buffer. Fractions of 1 ml were collected from the column, and the concentration of DOTA‐trastuzumab or NODAGA‐trastuzumab was determined by HPLC and Nanodrop.

### MALDI‐TOF MS analysis

The average number of chelators per trastuzumab molecule was determined using MALDI‐TOF MS. Prior to analysis, the samples (2–4 µg) were micro‐purified using an in‐house constructed micro‐column. The column was prepared by constricting the end of a GELoader® pipette tip (Eppendorf) and applying POROS® R1 20 resin (Applied Biosystems, Life Technologies) to an approximate height of 25 mm. For loading the sample and washing, 0.1% formic acid in water was used. The sample was eluted from the column with 0.1% formic acid, 80% acetonitrile in water and lyophilised to dryness. A thin layer of a saturated sinapinic acid solution in ethanol was applied onto the target plate (Bruker Daltonik GmbH) and left to dry. On top of this layer was added a mixture (50:50 *v*/*v*) of the dry sample dissolved in 0.1% TFA and a saturated sinapinic acid solution in 0.1% TFA, 30% acetonitrile in water. The samples were left to dry before the analysis was performed.

### Radiochemistry

[^64^Cu]CuCl_2_ (2000 MBq) was received in dry form and was dissolved in 0.1 M NaOAc pH 5.64 (1–2 ml). Reactions were performed in LoBind tubes from Eppendorf.

#### ^64^Cu‐labelling experiments

To a solution of DOTA‐trastuzumab/NODAGA‐trastuzumab (25 µg) in 0.1 M NaOAc (pH 5.64) was added ^64^Cu (50 µl, 50–100 MBq) and 0.1 M NaOAc (pH 5.64) to give a finale volume of 300 µl. The reaction was left at room temperature. Samples were taken after 15, 30, 60 and 90 min and analysed by radioHPLC and TLC (ScanRam). The samples were quenched with EDTA (0.1 M in phosphate buffer pH 7) prior to analysis to chelate free ^64^Cu ions for ease of analysis.

^64^Cu‐incorporation was studied by varying the amount of DOTA‐trastuzumab used (2–25 µg). Reactions were performed at either room temperature or 40°C.

#### Synthesis of [^64^Cu]DOTA‐trastuzumab

DOTA‐trastuzumab (100 µg) was labelled with ^64^Cu (250 MBq) in 0.1 M NaOAc (pH 5.64) at room temperature for 30 min. The product was purified by size exclusion chromatography with a PD‐10 column using phosphate‐buffered saline (PBS). [^64^Cu]DOTA‐trastuzumab was obtained with a radiochemical purity of 97.5% and a specific radioactivity of 225 GBq/µmol. The activity of the final product was 116 MBq.

#### Synthesis of [^64^Cu]NODAGA‐trastuzumab

NODAGA‐trastuzumab (100 µg) was labelled with ^64^Cu (200 MBq) in 0.1 M NaOAc (pH 5.64) at room temperature for 60 min. The product was purified by size exclusion chromatography with a PD‐10 column using PBS. [^64^Cu]NODAGA‐trastuzumab was obtained with a radiochemical purity of 97.7% and a specific radioactivity of 225 GBq/µmol. The activity of the final product was 54 MBq.

### *In vivo* stability of [^64^Cu]‐labelled trastuzumab

Blood samples (30 µl) were drawn from the tail vein of 2 mice, 1 injected with [^64^Cu]DOTA‐trastuzumab and 1 injected with [^64^Cu]NODAGA‐trastuzumab, 16 h post injection. EDTA (30 µl) was added to the samples, and the samples were analysed by TLC using a Packard Instant Imager.

### Cell culture and animal model

SK‐OV‐3 ovarian adenocarcinoma cells (ATCC HTB‐77, LGC Standards) were cultured in McCoy's 5A Medium Modified supplemented with 10% fetal bovine serum and 1% penicillin‐streptomycin (Invitrogen) at 37°C and 5% CO_2_. Cells in their exponential growth phase were harvested by trypsinization and suspended at 1 × 10^8^ cell/ml in 1:1 complete medium and matrigel (BD Biosciences). Eight weeks old female NMRI nude mice were inoculated with two tumours each. Fifty microlitres (5 × 10^6^ cells) of the cell suspension were injected with a 27G needle into the fourth mammary fat pad and subcutaneously into the flank above the hind limb. Tumours were allowed to grow for 4 weeks before imaging experiments. All animal procedures were performed under a protocol approved by the National Animal Experiments Inspectorate.

#### Saturation binding analysis

HER2‐positive SK‐BR‐3 breast adenocarcinoma cells (ATCC HTB‐30, LGC Standards) were cultured in McCoy's 5A Medium Modified supplemented with 10% fetal bovine serum and 1% penicillin‐streptomycin (Invitrogen) at 37°C and 5% CO_2_. Cells were seeded at 5 × 10^4^ cells per well in a 96‐transwell plate (Corning) and allowed to attach for 48 h. Cells were washed with PBS and incubated on ice for 3 h with 0.07‐150 nM [^64^Cu]DOTA‐trastuzumab or [^64^Cu]NODAGA‐trastuzumab in PBS with 1% human serum albumin and 0.1% sodium azide. Measurements were performed in triplicate, and non‐specific binding was estimated by incubating with 100‐fold excess of trastuzumab. Cells were washed 3 times with PBS, and the membrane inserts were dried at 65°C for 15 min. Membranes were transferred to tubes, and the cell bound activity was measured in a well counter (Wizard^2^, PerkinElmer). The K_D_ values were calculated based on a non‐linear regression model that compensates for non‐specific binding (one site–total and non‐specific binding, Prism 6.0d, GraphPad Software Inc.).

#### Small animal PET/CT imaging

*In vivo* small animal PET/CT imaging was performed on an Inveon Multimodalty PET/CT scanner (Siemens). Mice were injected intravenously by the tail vein with an average of 15.5 MBq (14.8–16.1 MBq) [^64^Cu]DOTA‐trastuzumab or 11.6 MBq (11.3–12.0 MBq) [^64^Cu]NODAGA‐trastuzumab in PBS. Mice were under gas anesthesia (1.5 l/min, 4% Sevoflurane in 35% O_2_ 65% N_2_) during injections and imaging sessions. A 10‐min PET acquisition was performed at 0.75, 13 and 26‐h post injection and a 20‐min PET acquisition 43‐h post injection. CT imaging was performed after each PET acquisition with the following settings: two bed positions, 360° rotation, 240 rotation steps, 400 ms exposure at 500 μA and 70 kV. CT images were reconstructed with an isotropic voxel size of 0.21 mm. PET images were reconstructed with CT based attenuation correction using the 3D maximum a posteriori algorithm, a voxel size of 0.42 × 0.42 × 0.80 mm and a spatial resolution at the centre of the field of view of 1.2 mm.

## Results and discussion

### Chelator conjugation

**Figure 1:**
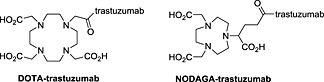
Structure of DOTA‐trastuzumab and NODAGA‐trastuzumab.

Trastuzumab was conjugated with either DOTA or NODAGA (Figure 1). The chelators were available as activated esters containing a succinimide, facilitating conjugation with lysine residues on the antibody. Excess chelator was removed by size exclusion chromatography using PD‐10 desalting columns, and fractions of 1 ml were collected. Typically, antibody eluted from fraction 3 to 6, and unbound chelator eluted around fraction 10. The fractions were analysed by HPLC, and the concentration was determined by absorbance at 280 nm using Nanodrop. This method could not, however, distinguish between trastuzumab and chelator conjugated trastuzumab.

### Average number of chelator per antibody

DOTA and NODAGA conjugates using 5, 20 and 100‐fold excesses of the reagents compared with trastuzumab were analysed using MALDI‐TOF MS analysis. The spectrum of conjugated antibody was compared with the spectrum of pure antibody in order to determine the mass difference and thus estimate the number of chelators per antibody molecule. The mass difference was calculated based on the doubly charged ion (approximately 75 000 Da), as the resolution and mass accuracy are higher than for the singly charged ion. The results for all conjugations with DOTA and NODAGA are shown in Table 1 (for spectra refer to the supporting information). As expected, the number of chelators conjugated was higher when a larger excess of chelator was used for the conjugation. For the DOTA conjugation only, a slightly higher number of chelators was seen for 100‐fold excess compared with 20‐fold excess. For NODAGA conjugation, the average number of chelators per antibody was lower for 5‐fold and 20‐fold excess compared with the DOTA, whereas it was higher for 100‐fold excess with an average of 27 NODAGA per trastuzumab. The number of DOTA chelator per trastuzumab molecule for the 5‐fold excess conjugation corresponds with the reported number of 1.7 when conjugation was performed using 3‐fold excess.^8^ For conjugation using 20 and 80 equivalents of DOTA, 6 and 5.8 chelator per trastuzumab were reported.^24^, ^26^ This is somewhat lower than our findings; however, these determinations were not performed using MALDI‐TOF MS.

**Table 1:** Average number of chelators per trastuzumab molecule, calculated based on the doubly charged molecular ion

	Equivalents of chelator used in conjugation		
	5	20	100
DOTA‐trastuzumab	2.9	11.2	12.7
NODAGA‐trastuzumab	1.4	7.3	26.6

### ^64^Cu‐labelling of trastuzumab

**Figure 2:**
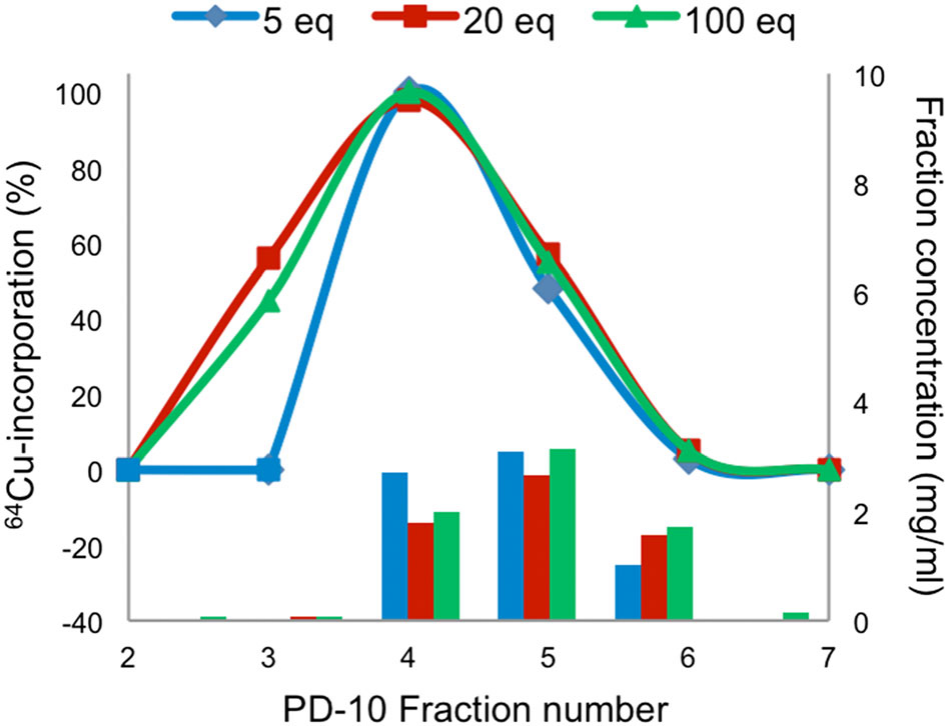
^64^Cu‐incorporation of DOTA‐trastuzumab in antibody containing fractions collected from PD‐10 purification after DOTA conjugation with 5, 20 and 100 equivalents of DOTA‐NHS ester. The bars show the antibody concentration in the fractions. Twenty‐five microgram DOTA‐trastuzumab and a total volume of 300 µl were used for all experiments. Reactions were performed at room temperature and analysed after 15 min.

The first DOTA conjugation to trastuzumab was performed with 5 equivalents of DOTA‐NHS ester. The concentration of antibody was determined in the fractions collected from PD‐10 purification using Nanodrop, and antibody was found in fractions 4 to 6. It was discovered that ^64^Cu‐incorporation varied depending on which antibody fraction was used for labelling. All three fractions (4–6) were labelled with copper‐64 using the same concentration of antibody. Full ^64^Cu‐incorporation was only seen for fraction 4 (Figure 2, blue), which suggests that DOTA was not conjugated to all trastuzumab molecules. The amount of DOTA‐NHS ester for conjugation to trastuzumab was then increased to 20 and 100. After purification to remove non‐reacted DOTA‐NHS ester, all fractions collected with antibody were labelled with copper‐64. The same mass of antibody and total volume were used to obtain the same concentration for labelling. The ^64^Cu‐incorporation varied in the different fractions (Figure 2, red and green), and full incorporation was seen in fraction 4 for all conjugations. The incorporation was determined after 15 min and did not increase further by longer reaction times. Due to these variations in ^64^Cu‐incorporation, it is likely that DOTA may not be conjugated to all trastuzumab molecules even though a high excess of DOTA‐NHS ester was used. Full incorporation was achieved in fraction 4 for all conjugations, which suggests that DOTA‐conjugated trastuzumab is eluting earlier from the PD‐10 column compared with trastuzumab. Some trastuzumab molecules might also be conjugated with more DOTA chelators compared with others, and these could possibly elute earlier.

**Figure 3:**
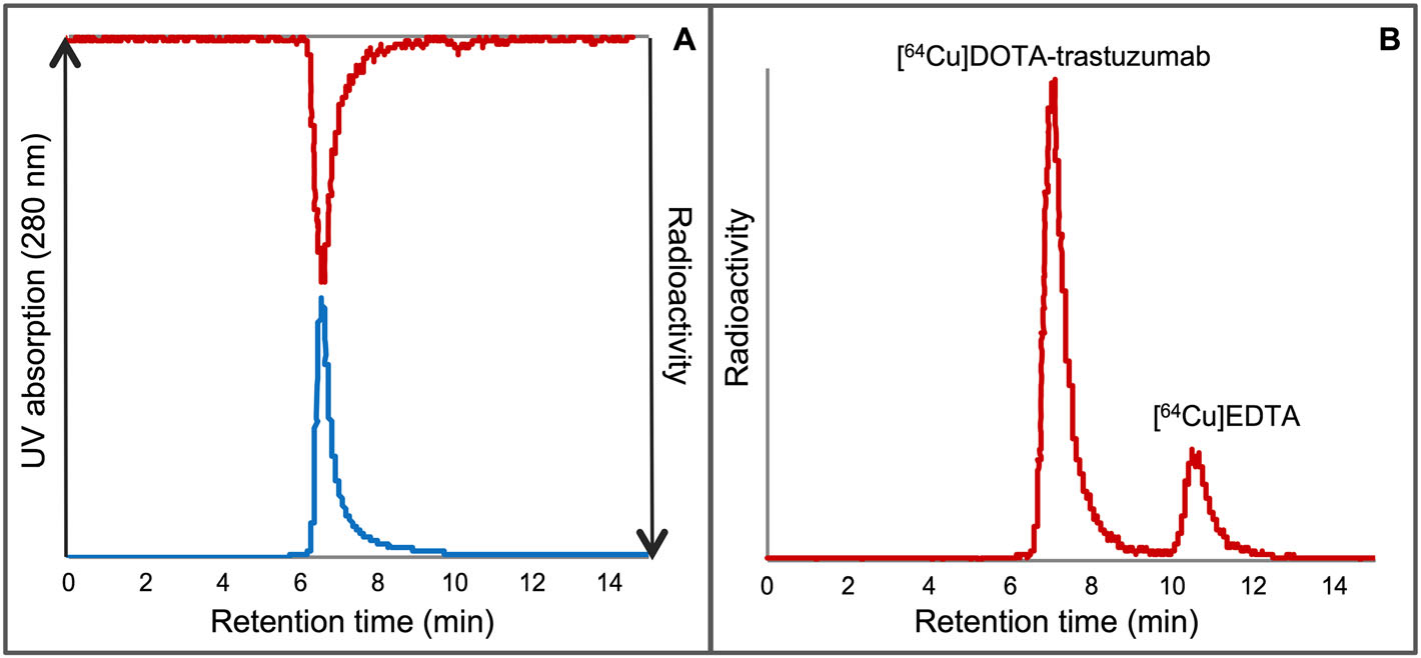
HPLC chromatogram of DOTA‐trastuzumab (blue) with overlay of [^64^Cu]DOTA‐trastuzumab (red) (A) and a crude mixture of [^64^Cu]DOTA‐trastuzumab with [^64^Cu]EDTA (B).

Chelator conjugation and labelling experiments were analysed by size exclusion HPLC. A chromatogram of DOTA‐trastuzumab with overlay of [^64^Cu]DOTA‐trastuzumab is shown in Figure 3, A. All samples were quenched with EDTA prior to analysis (HPLC and TLC) in order to chelate free copper‐64. [^64^Cu]EDTA eluted around 11 min in the HPLC analysis (Figure 3b). HPLC results were confirmed by radioTLC analysis, where ^64^Cu‐labelled trastuzumab had a Rf value of 0 and [^64^Cu]EDTA 0.9.

^64^Cu‐labelling of trastuzumab was discovered to be quite sensitive. The dried copper‐64 was received in a glass container and was dissolved in a sodium acetate buffer prior to the labelling reactions. Incorporation using the same mass of DOTA‐trastuzumab varied depending on when the reaction was performed. Especially, labelling reactions performed the day after receiving copper‐64 gave low incorporation. However, labelling reactions were consistent when copper‐64 was transferred from the glass container to a plastic container after it was dissolved. The glass container may release metal ions that bind to the chelator and thus prevent binding of copper‐64. The glass containers have, however, not been an issue for copper‐64 experiments performed on other molecules at our facility.

**Figure 4:**
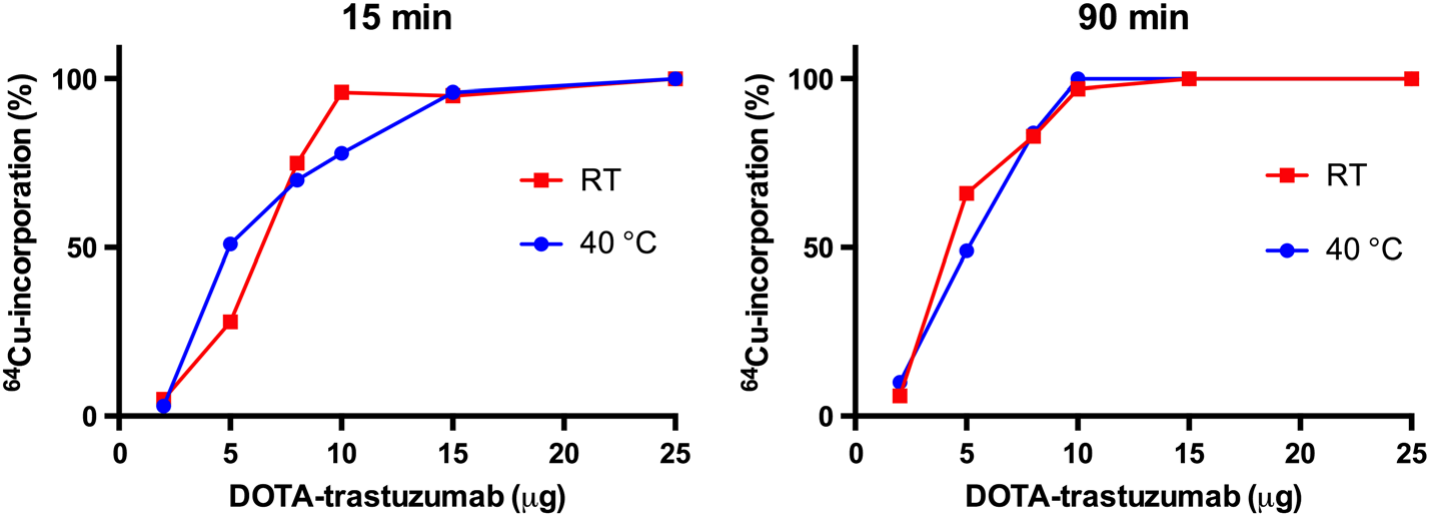
Incorporation curves for the ^64^Cu‐labelling of DOTA‐trastuzumab using different amounts of DOTA‐trastuzumab. Reactions were performed at room temperature (blue) and at 40°C (green) and analysed at different times: 15 (A) and 90 min (B) are shown. The volume was held constant in all experiments.

Conditions for ^64^Cu‐labelling of DOTA‐trastuzumab were studied by varying the amount of DOTA‐trastuzumab used, reaction temperature and time. The ^64^Cu‐incorporation at different reaction times and temperatures is shown in Figure 4. Initially, the labelling was tested with 25–200‐µg DOTA‐trastuzumab, and full ^64^Cu‐incorporation was achieved after 15 min at room temperature. It was decided to study the ^64^Cu‐incorporation at 2–25 µg of DOTA‐trastuzumab. For all time points and temperatures, full ^64^Cu‐incorporation was achieved for 25‐µg DOTA‐trastuzumab. The incorporation was more than 95% at all times when using 15‐µg DOTA‐trastuzumab. Increasing the temperature to 40°C gave full incorporation after 30 min. For 10‐µg DOTA‐trastuzumab, the incorporation was higher at 40°C with full incorporation after 30 min. Full incorporation was not seen when using 5‐ or 7.5‐µg DOTA‐trastuzumab, and the incorporation was similar at both temperatures. For 2‐µg DOTA‐trastuzumab, ^64^Cu‐incorporation was low with 10% as the highest. Full ^64^Cu incorporation could be achieved using more than 10‐µg DOTA‐trastuzumab. Optimal labelling conditions were obtained with 25‐µg DOTA‐trastuzumab and labelling at room temperature for 15 min. These conditions were an improvement compared with previously published reports.^8^, ^24^, ^25^, ^26^ Ferreira *et al*. reported a ^64^Cu‐incorporation of 88% using 0.5‐mg DOTA‐trastuzumab after 2 h at room temperature, whereas only 25% incorporation was observed when using 0.1 mg.^8^ In another study, 30‐µg DOTA‐trastuzumab was labelled with copper‐64 at 40°C for 1.5 h with an incorporation of 92%.^24^ In the human study with [^64^Cu]DOTA‐trastuzumab, labelling was achieved after 1 h at 40°C with 98% incorporation.^25^ The mass of DOTA‐trastuzumab used for labelling was not reported, but based on the reported specific radioactivity of 350 GBq/µmol and activity of the final product, an estimated mass of 200‐µg DOTA‐trastuzumab was used. Alirezapour *et al*. also labelled DOTA‐trastuzumab with copper‐64.^26^ Incorporation was higher than 90% using 250‐µg DOTA‐trastuzumab and labelling at 40°C for 1 hour. Our results are an improvement with respect to the amount of DOTA‐trastuzumab required for full incorporation and also reaction time and temperature.

**Figure 5:**
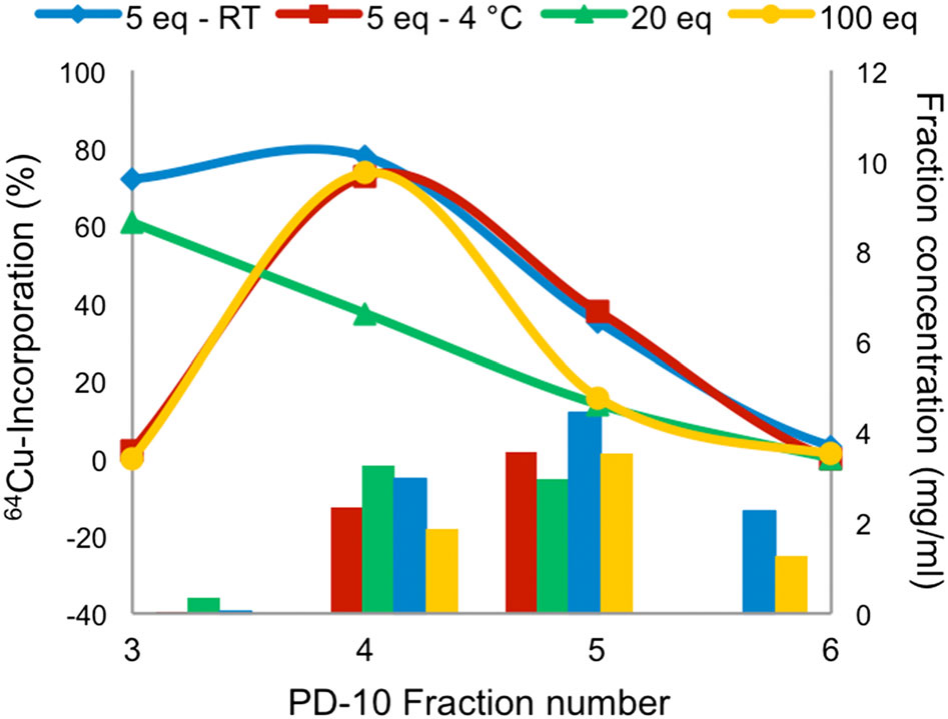
^64^Cu‐incorporation of NODAGA‐trastuzumab in antibody containing fractions collected from PD‐10 purification after NODAGA conjugation with 5, 20 and 100 equivalents NODAGA‐NHS ester. Conjugation was also tested with 5 equivalents NODAGA‐NHS ester at room temperature (5 equivalents—RT, blue). The bars show the antibody concentration in the fractions. Twenty‐five microgram NODAGA‐trastuzumab and a total volume of 300 µl were used for all experiments. Reactions were performed at room temperature and analysed after 15 min.

^64^Cu‐labelling of trastuzumab was also investigated using NODAGA as chelator. NODAGA conjugation was studied using different amount of NODAGA‐NHS ester (5, 20 and 100 equivalents). The conjugation with 5 equivalents was also performed at room temperature with constant shaking overnight. All antibody containing fractions from all three conjugations were labelled with copper‐64 at room temperature using the same mass of NODAGA‐trastuzumab. The ^64^Cu‐incorporation for the different fractions and conjugations after 15 min at room temperature is shown in Figure 5. Full incorporation was not seen for any of the fractions, and the incorporation did not increase by longer reaction times or by increasing the temperature to 40°C. The labelling reactions were performed using 25‐µg NODAGA‐trastuzumab, which previously was found to be adequate for DOTA‐trastuzumab. Increasing the mass of NODAGA‐trastuzumab did not increase the ^64^Cu‐incorporation. Conjugation with 5 equivalents of NODAGA‐NHS at room temperature had high ^64^Cu‐incorporation for both fractions 3 and 4, which was not seen for the conjugation performed at 4°C. The highest incorporation (80%) was achieved using fraction 4, and this fraction was also found to give the best results for DOTA‐trastuzumab labelling. [^64^Cu]NODAGA‐trastuzumab could be purified with a radiochemical purity >97% using PD Minitrap columns and collecting fractions of 0.2 ml. Collecting a higher volume gave fractions with overlap of [^64^Cu]NODAGA‐trastuzumab and free copper‐64. The average number of NODAGA per trastuzumab was lower than for DOTA for 5 and 20 equivalents conjugations. This could be a reason for the lower incorporation. However, conjugation with 100‐fold NODAGA excess had the highest amount of chelator per trastuzumab, but still did not give full incorporation. These results could indicate that the chelators are conjugated at sites that may be sterically hindered and thus not available for labelling. Higher incorporation would be expected for antibody molecules containing a larger amount of chelators.

For *in vitro* and *in vivo* studies, both chelators were conjugated using 5 equivalents giving 2.9 DOTA per antibody and 1.4 NODAGA per antibody. Labelling of DOTA‐trastuzumab and NODAGA‐trastuzumab was tested with higher amounts of copper‐64 to prepare the synthesis for *in vivo* studies. ^64^Cu‐incorporation in both compounds was lower than previously seen. The mass of DOTA‐trastuzumab and NODAGA‐trastuzumab was therefore increased to 100 µg in order to achieve higher incorporation. Labelling did not give full ^64^Cu‐incorporation and both compounds required purification using the larger PD‐10 columns. Purification using MiniTrap columns led to incomplete separation of product from free copper‐64. [^64^Cu]DOTA‐trastuzumab and [^64^Cu]NODAGA‐trastuzumab were synthesised with radiochemical purities of 97.5 and 97.7% respectively, and specific radioactivity of ~225 GBq/µmol. Chelator conjugated trastuzumab solutions were prepared and stored at −80°C a few weeks before labelling with copper‐64. The reason for the lower copper‐64 incorporation has not been fully studied, and it is thus not clear whether this is caused by the higher amounts of radioactivity (and thus higher carrier copper content), or the instability of the conjugates on storage and subsequent thawing.

### Saturation binding analysis

**Figure 6:**
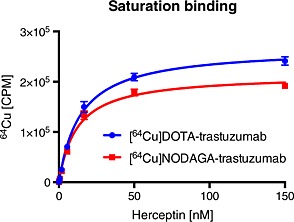
Saturation binding of [^64^Cu]DOTA‐trastuzumab and [^64^Cu]NODAGA‐trastuzumab to SK‐BR‐3 cells. The K_D_ values were estimated to 14.1 and 12.0 nM respectively.

The affinities of [^64^Cu]DOTA‐trastuzumab and [^64^Cu]NODAGA‐trastuzumab in the cell based saturation binding assay were estimated to 14.1 nM (95% CI, 12.1–16.0 nM) and 12.0 nM (95% CI, 10.1–13.8 nM) respectively (Figure 6). Chelator conjugation and radiolabelling did not have a large effect on the affinity compared with the reported affinity of trastuzumab in a cell‐based assay of 5.0 nM.^27^

### MicroPET imaging

**Figure 7:**
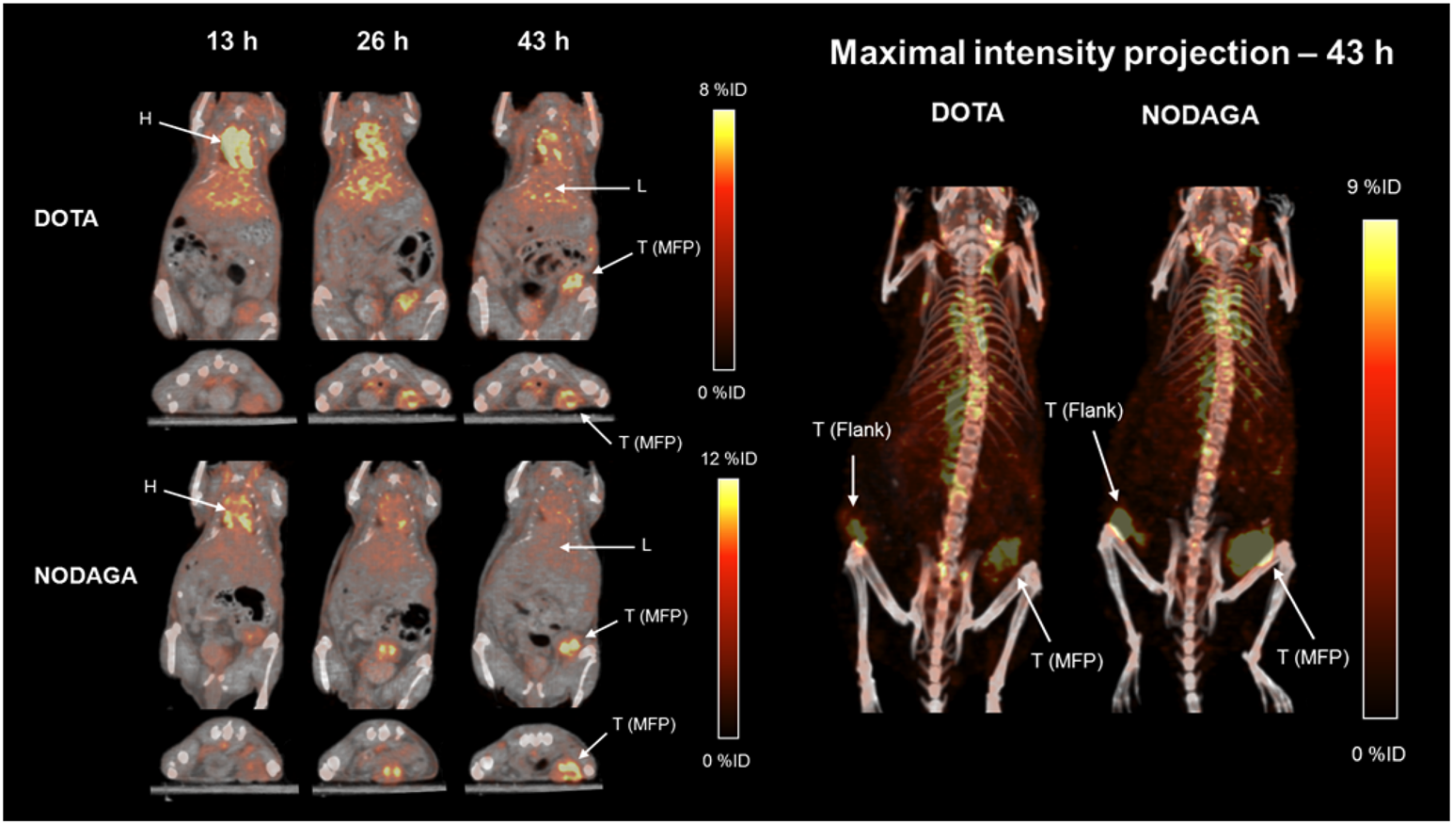
Coronal and axial PET/CT images of [^64^Cu]DOTA‐trastuzumab (DOTA) and [^64^Cu]NODAGA‐trastuzumab (NODAGA) 13, 26 and 43‐h post injection (left), and maximal projection images 43‐h post injection (right). H, heart; L, liver; T, tumour; MFP, mammary fad pad.

[^64^Cu]DOTA‐trastuzumab and [^64^Cu]NODAGA‐trastuzumab were injected into two mice each with two HER2 expressing tumours in the mammary fad pat and flank respectively. The mice were imaged by PET 13, 26 and 43‐h post injection (Figure 7). Both tracers showed good tumour uptake which increased uptake over time; [^64^Cu]DOTA‐trastuzumab: mammary fad pad 3.49% ID/g (13 h) to 5.98% ID/g (43 h), flank 3.28% ID/g (13 h) to 5.62% ID/g (43 h), [^64^Cu]NODAGA‐trastuzumab: mammary fad pad 4.78% ID/g (13 h) to 8.32% ID/g (43 h), flank 4.42% ID/g (13 h) to 8.81% ID/g (43 h).

In this preliminary evaluation, [^64^Cu]NODAGA‐trastuzumab proved to be a promising tracer for HER2 imaging. Since copper‐64 is known to dissociate from DOTA *in vivo*, it was desirable to find a better chelator. Further *in vivo* studies with [^64^Cu]‐labelled trastuzumab are necessary to determine whether NODAGA is better than DOTA. Very recently, the chelators DOTA and NODAGA were compared in ^64^Cu‐labelling of an immunoconjugate.^28^ The labelled compounds were evaluated in mice with PC‐3 tumours and subjected to biodistribution studies. The results suggested that NODAGA is more stable *in vivo* and thus preferred over DOTA.

### *In vivo* stability

A TLC method to separate free copper‐64 and [^64^Cu]‐labelled trastuzumab was developed. The stability of the two tracers were studied by analysing blood samples from the two mice injected with either [^64^Cu]DOTA‐trastuzumab or [^64^Cu]NODAGA‐trastuzumab. The samples were drawn 16 hours post injection and analysed by TLC. TLC analysis showed no free copper‐64, indicating high *in vivo* stability for both compounds.

## Conclusions

^64^Cu‐Labelling of DOTA‐trastuzumab was optimised with respect to amounts of DOTA‐trastuzumab used, reaction temperature and time. Full ^64^Cu‐incorporation could be achieved using 10‐µg DOTA‐trastuzumab. The fastest labelling conditions were obtained after 15 min at room temperature using 25‐µg DOTA‐trastuzumab. Labelling of trastuzumab was also for the first time studied with NODAGA as chelator. The ^64^Cu‐incorporation was not as high as for DOTA‐trastuzumab with 80% being the best obtained. However, [^64^Cu]NODAGA‐trastuzumab could be purified using PD MiniTrap columns giving a radiochemical purity of more than 97%. The two tracers were evaluated in mice with HER2 expressing tumours using PET imaging, and both tracers showed good tumour uptake in mice. Further *in vivo* studies will be performed in order to evaluate which of the two tracers is the optimal candidate for HER2 PET imaging.

## Conflict of Interest

The authors did not report any conflict of interest.

## Supporting information

Supporting info item
