# Supplementary material for: [64Cu]‐labelled trastuzumab: optimisation of labelling by DOTA and NODAGA conjugation and initial evaluation in mice
Source: J Labelled Comp Radiopharm. 2015 Apr 24;58(6):227–33. doi: 10.1002/jlcr.3287 (PMC5029596; doi:10.1002/jlcr.3287)
Supplement: Supplementary file 1 — Supporting info item [file JLCR-58-227-s001.docx]

Supporting information

MALDI spectra of chelator conjugated trastuzumab. Chelator was conjugated in 5, 20 or 100-fold excess compared to trastuzumab.

**Trastuzumab**

**DOTA-trastuzumab, 5 equivalents chelator**

**DOTA-trastuzumab, 20 equivalents chelator**

**DOTA-trastuzumab, 100 equivalents chelator**

**NODAGA-trastuzumab, 5 equivalents chelator**

**NODAGA-trastuzumab, 20 equivalents chelator**

**NODAGA-trastuzumab, 100 equivalents chelator**
